# Can We Compare the Health-Related Quality of Life of Childhood Cancer Survivors Following Photon and Proton Radiation Therapy? A Systematic Review

**DOI:** 10.3390/cancers14163937

**Published:** 2022-08-15

**Authors:** Mikaela Doig, Eva Bezak, Nayana Parange, Peter Gorayski, Victoria Bedford, Michala Short

**Affiliations:** 1 Allied Health and Human Performance Academic Unit, University of South Australia, Adelaide, SA 5000, Australia; 2 Icon Cancer Centre Windsor Gardens, Adelaide, SA 5087, Australia; 3 Department of Physics, University of Adelaide, Adelaide, SA 5000, Australia; 4 Department of Radiation Oncology, Royal Adelaide Hospital, Adelaide, SA 5000, Australia; 5 Australian Bragg Centre for Proton Therapy and Research, Adelaide, SA 5000, Australia; 6 Cancer Voices South Australia, Adelaide, SA 5068, Australia

**Keywords:** quality of life, radiation oncology, paediatric oncology, proton therapy, survivorship, patient-reported outcomes

## Abstract

**Simple Summary:**

Proton radiation therapy is a radiation oncology innovation expected to produce superior health-related quality of life (HRQoL) outcomes for children with cancer, compared to conventional photon radiation therapy. The review aim is to identify if clinical evidence exists to support the anticipated HRQoL improvements for children receiving proton radiation therapy. HRQoL outcomes of 1986 childhood cancer survivors are described. There is insufficient quality evidence to compare HRQoL outcomes between proton and photon radiation therapy. Therefore, the current state of the literature does not conclude that proton radiation therapy produces superior HRQoL outcomes for childhood cancer survivors. Despite recommendations, no evidence of routine HRQoL assessment using patient-reported outcomes in paediatric radiation oncology are identified. Further rigorous collection and reporting of HRQoL data is essential to improve patient outcomes, and to adequately compare HRQoL between radiation therapy modalities.

**Abstract:**

Paediatric cancer patients have a risk of late side effects after curative treatment. Proton radiation therapy (PRT) has the potential to reduce the incidence and severity of toxicities produced by conventional photon radiation therapy (XRT), which may improve the health-related quality of life (HRQoL) in children. This systematic review aimed to identify the evidence of HRQoL outcomes in childhood cancer survivors following XRT and PRT. Medline, Embase, and Scopus were systematically searched. Thirty studies were analysed, which described outcomes of 1986 childhood cancer survivors. Most studies (*n* = 24) described outcomes for children with a central nervous system (CNS) tumour, four studies reported outcomes for children with a non-CNS tumour, and two studies combined CNS and non-CNS diagnoses within a single cohort. No studies analysed routine HRQoL collection during paediatric radiation oncology clinical practice. There is insufficient quality evidence to compare HRQoL outcomes between XRT and PRT. Therefore, the current state of the literature does not conclude that PRT produces superior HRQoL outcomes for childhood cancer survivors. Standardised clinical implementation of HRQoL assessment using patient-reported outcomes is recommended to contribute to improvements in clinical care whilst assisting the progression of knowledge comparing XRT and PRT.

## 1. Introduction

Radiation therapy (RT) is used to provide treatment for paediatric cancers, often in combination with surgery, chemotherapy, and/or immunotherapy. Whilst multimodal curative intent treatments increase the probability of cures, it comes with an added risk of late side effects [1]. Common RT-induced late-presenting toxicities include neurocognitive effects, psychosocial effects, endocrine abnormalities, and second primary cancer development [1,2,3,4,5]. Children with cancer are treated during physical and cognitive development and are, therefore, more susceptible to developmental and functional impairment than adults, whilst having the additional burden of a lifetime to experience these chronic late effects [6].

Late effects of RT are associated with poor health-related quality of life (HRQoL) [3,5]. HRQoL assessment of childhood cancer survivors includes domains of physical functioning (e.g., impact of pain and symptoms), psychosocial functioning (e.g., self-esteem and body image), cognitive functioning (e.g., school performance), and social functioning (e.g., interpersonal relationships) [7]. Due to the highly subjective nature of these domains, patient-reported outcomes are used to gain experiential insights and direct symptomatic reports from the patient [8,9].

Proton radiation therapy (PRT) is increasingly used in paediatric radiation oncology [10]. The unique absorbed dose deposition of charged particles can reduce the volume of healthy tissues receiving medium to low radiation doses, and, therefore, has the potential to reduce the risk of long-term toxicities and second primary cancer induction when compared to photon radiation therapy (XRT) [11,12]. PRT is not as widely available, with many countries lacking access to this modality [13], so XRT remains the standard of care for many service providers delivering RT to children with cancer.

Radiobiological modelling and initial clinical evidence demonstrate the advantages of using PRT to improve health outcomes in paediatric cancer patients [11,14,15]; however, the evidence comparing XRT and PRT HRQoL outcomes in childhood cancer survivors has not been previously synthesised. Additionally, despite recognition of patient-reported HRQoL assessment as a meaningful outcome for children with cancer, it is unknown if reported HRQoL outcomes are used in routine clinical care for paediatric RT patients. Therefore, this systematic review aimed to (1) identify and evaluate the current evidence and utilisation of patient-reported HRQoL assessment in childhood cancer survivors following XRT and PRT, and (2) to determine if PRT results in improved HRQoL outcomes for childhood cancer survivors.

## 2. Materials and Methods

### 2.1. Search Strategy

This systematic review was conducted in accordance with preferred reporting items for systematic reviews and meta-analysis (PRISMA) 2020 guidelines [16]. The review protocol was registered with the international prospective register of systematic reviews (PROSPERO, registration number: CRD42021249369). Medline, Embase, and Scopus were systematically searched from 1 January 2000 to 12 May 2021, to identify all articles describing the patient-reported quality of life in childhood cancer survivors following XRT or PRT (see Supplemental Table S1 for the search strategy). The search was updated on 1 May 2022, with no further eligible studies identified by the first author.

### 2.2. Selection Criteria

Studies were included if they were peer-reviewed, original research articles published in the English language, if a validated patient-reported outcomes tool was used to measure quality of life, and if participants were children diagnosed with cancer between ages 0 to 21 years [17]. Studies were eligible if participants received external beam RT (XRT or PRT), with total body irradiation excluded, as a comparable technique using particle therapy is not in clinical use [18]. If an analysis of a patient cohort included patients with and without RT treatment, 50% or more participants were required to have received RT. Studies were excluded if RT was delivered prior to the year 2000, as the resulting patient outcomes may not reflect contemporary modulated treatment planning and delivery techniques. If a longitudinal analysis included patients treated prior to 2000, the article needed to provide sufficient details to confirm that the treatment delivery method was still used by the treating facility as current practice, or include an analysis by decade.

### 2.3. Screening and Data Extraction

**Figure 1:**
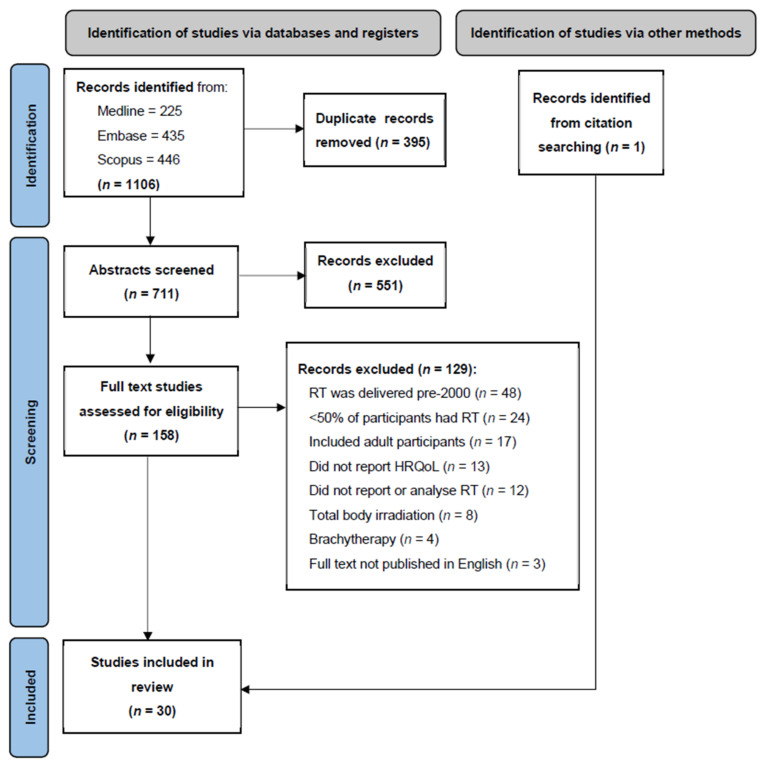
Preferred reporting items for systematic reviews and meta-analyses (PRISMA) flow diagram detailing selection process and exclusion reasons. Abbreviations: RT, radiation therapy; HRQoL, health-related quality of life.

Two authors (MD and MS) performed independent screening of title and abstracts after removal of duplicate records, followed by full-text assessment for inclusion. In situations where agreement for inclusion could not be reached, consensus was achieved by inviting additional authors for decision making (EB and NP). Figure 1 summarises the screening process, including exclusion reasons. Author MD performed reference and citation searching of the included studies (pearling), which identified one additional study. Author MD extracted all eligible data from the included studies. Author MS extracted data from three studies to confirm reliable extraction. If multiple studies reported results for the same patient cohort, the study with greater follow up was included. Studies that included a potentially overlapping cohort of patients, but provided different analyses, were included as separate records but outcomes were summarised together in Table 1, Table 2, Table 3 and Table 4.

### 2.4. Quality Assessment and Analysis

Two authors (MD and MS) performed quality assessment for all included studies using the 14 item QualSyst quantitative checklist [19] (Supplemental Table S2). Each criterion was assessed by compliance with the corresponding recommendations in ‘International standards for the analysis of quality of life and patient-reported outcome endpoints in cancer randomised controlled trials’ [20]. An agreement of 94.85% was observed between the two authors. Studies were not excluded based on a quality assessment threshold. Studies that used the Pediatric Quality of Life Inventory (PedsQL) Generic Core (Version 4.0) patient-reported outcome measure and provided the total summary score (total core score) of the cohort were included for further analyses. PedsQL is a validated, 23 item questionnaire, designed to measure HRQoL in children and adolescents via self-reporting and parent–proxy reporting [21]. Modules are grouped into four domains (physical, emotional, social, and school functioning), which are averaged to produce a ‘total core’ summary score, scaled from 0 to 100, with higher scores indicating better HRQoL [21]. Studies were included in the further analysis if they provided a measure of central tendency and a measure of dispersion or variation, or the raw data. A biostatistician was consulted to advise on the use of meta-analytic methods; however, due to heterogeneity across published data, a meta-analysis was not possible. Overall data were tabulated, grouped by tumour site (central nervous system (CNS) vs. non-CNS), and RT modality. Studies were grouped in tumour sites and ordered by quality assessment score. Comparisons were made between modality and tumour sites where applicable.

## 3. Results

### 3.1. Study Characteristics

Table 1, Table 2, Table 3 and Table 4 summarise 30 studies that meet the inclusion criteria for review, reporting outcomes of 1986 childhood cancer survivors. A total of 14 studies are cross-sectional, 15 studies are longitudinal, and 1 study uses both longitudinal and cross-sectional data collection. Articles are included from the United States [22,23,24,25,26,27,28,29], Switzerland [30,31,32,33], South Korea [34,35], Canada [36,37], Japan [38,39], Netherlands [40,41], India [42], United Kingdom [43,44], Germany [45], Brazil [46], Italy [47], Poland [48], North American collaboration [49], European collaboration, [50] and an international collaborative [51].

A total of 19 studies analyse HRQoL after XRT, and 10 studies analyse HRQoL after PRT. One study includes survivors treated with XRT or PRT [45]. Yock et al. (2014) compare the HRQoL in two cohorts from separate institutions, one treated with PRT and one treated with XRT [25]. Only the PRT results from this study were included in this review, as the XRT data do not meet the inclusion criteria. Dessens et al. (2016) describe two cohorts following XRT, with only the cohort attending follow up in April 2014 meeting inclusion criteria [41].

### 3.2. Central Nervous System Cancers

Twenty-four articles capture HRQoL in survivors of paediatric CNS tumours following RT (see Table 1 and Table 2). A total of 11 studies analyse individual diagnoses, whilst 12 combine several different CNS diagnoses within their analyses. Eight studies analyse the HRQoL in patients diagnosed with a tumour of the brainstem or cerebellum (including medulloblastoma [22,43,47,50,51], diffuse intrinsic pontine glioma [28,40], or atypical teratoid rhabdoid tumour [32]. Kamran et al. (2018) describe self-reported increases in HRQoL annually for a median of 5 years post-PRT [22], whilst Veldhuijzen van Zanten et al. (2017) survivors self-reported worsening in nausea and fear of procedures at 3 months post-XRT [40]. However, the other longitudinal studies identify no significant changes to self-reported HRQoL with time [28,32,43]. Two studies use PedsQL Generic Core score to evaluate HRQoL in survivors of childhood retinoblastoma [23,42]. HRQoL scores of both cohorts do not significantly differ from the normative PedsQL reference population scores [52].

**Table 1:** Health-related quality of life in survivors of central nervous system tumours treated with photon radiation therapy.

Author	Diagnosis and Sample	Age at Diagnosis (Years)	Treatment Regimen	*n* (%) with RT	RT Modality and Technique	Prescribed Dose	PROM	Assessment Point(s)	Statistically Significant Outcomes
deMedeiros et al. (2020) [51]	Medulloblastoma *n* = 76	Mean (SD) 6.71 (3.56)	Surgery (GTR: 85.1%), CT (98.7%), and/or XRT (97.3%)	74 (97.3)	XRT CSI + PFB or tumour bed boost	30.6 to 39.4 Gy *n* = 24; 18.0 to 23.4 Gy *n* = 50	Health Utilities Index Mark 2 and 3 SR only: *n* = 13; PPR only: *n* = 36; SR + PPR: *n* = 27	Mean (SD) time from diagnosis: 6.58 (4.00) years	No difference in overall HRQoL between Western and Eastern countries. Higher proportion of PPR reporting moderate to severe morbidity burden than SR
Kennedy et al. (2014) [50]	Medulloblastoma *p* = 151; standard XRT (S) *n* = 77; hyper-fractionated XRT (H) *n* = 74	Median (range) S: 7.7 (3.3–20.4); H: 8.7 (3.2–20.8)	Surgery + concurrent ChemoRT + adjuvant CT. CT protocol was the same for S + H patients	151 (100)	XRT S: CSI + PFB H: CSI + PFB + tumour bed boost	S: 23.4 Gy CSI + 54 Gy PFB (30#, 1.8 Gy/#); H: 36 Gy CSI + 60 Gy PFB + 68 Gy tumour bed (60#, 1.0 Gy/# twice daily)	PedsQL Generic Core: PPR for participants aged < 18 years; SR for 11–17 EORTC quality of life measure (QLQ-C30): SR aged > 18	Median (range) time from diagnosis: S: 5.8 (4.1–9.8) years; H: 5.7 (4.2–9.9) years	No difference between SRT and HRT total scores for PedsQL or QLQ-C30
Bull et al. (2014) [43]	Medulloblastoma (M) *n* = 37; cerebellar astrocytoma (A) *n* = 35; matched control group (C) *n* = 38	Mean (range) M: 8.2 (6–13) A: 9.2 (5–14)	M: Surgery + XRT + Packer regimen CT; A: Surgery only	M: 37 (100) A + C: 0	XRT CSI + PFB	CSI: 23.4 Gy; PFB: 55.8 Gy	PedsQL Generic Core SR + PPR	T1: at recruitment (1–35 months from diagnosis) T2: 12 months post-T1 T3: 24 months post-T1	No change in SR or PPR overtime. HRQoL scores were lower for M than A cohort, and lower for A than C
Veneroni et al. (2017) [47]	Metastatic medulloblastoma *p* = 25 ^ *n* (completing PROM) = 14	Median (IQR) 10.8 (7.0–13.9)	Surgery + CT + XRT ± myeloablative CT	14 (100)	XRT Hyper-fractionated accelerated XRT strategy CSI + PFB ± metastases	CSI: 31.2–39 Gy; PFB: 59.7–60 Gy; metastases: additional 9 Gy Twice daily 1.3–1.5 Gy fractions	SR 12–17 years: PedsQL Generic Core SR 18+ years: PedsQL Generic Core + QLQ-30 + SF-36	Median (IQR) time from treatment: 12.6 (IQR 7.4–14.9) years	PedsQL and QLQ-30 scores do not differ from the general population. SF-36 psychological, physical, and mental scale scores were worse than the general population
Mandrell et al. (2016) [28]	Diffuse intrinsic pontine glioma *n* = 24; medullary glioblastoma *n* = 1	Median (range) 5.8 (2.3–17.2)	ChemoRT (Phase 1 clinical trial to determine maximum tolerated CT)	25 (100)	XRT Conformal *n* = 24; whole brain *n* = 1	54 Gy (*n* = 24), 55.8 Gy (*n* = 1)	PedsQL Generic Core: PPR <4 years, SR 5+ PedsQL brain tumour module: PPR all ages, SR 5+	T1: baseline pre-XRT T2: week 2 of XRT T3: week 4 of XRT T4: week 6 of XRT (last week of XRT) T5: 10 weeks post-XRT (week 16)	No change in SR PedsQL Generic Core subscales from T1–5 (total score not reported). PPR improvement from T1–2 in cognitive problems, movement and balance, procedural anxiety, and brain tumour module total score
Veldhuijzen van Zanten et al. (2017) [40]	Diffuse intrinsic pontine glioma *n* = 9	Median (range) 10.8 (7.5–17.3)	ChemoRT (Phase I/II clinical trial to determine maximum tolerated CT)	9 (100)	XRT Volumetric-modulated arc therapy	54 Gy	PedsQL: Generic Core, multi -dimensional fatigue scale, and cancer module SR	T1: baseline T2: 3 months post-XRT	Worsening in nausea (SR) and fear of procedures (SR + PPR) scales on cancer module at T2. No change from T1–T2 in generic or fatigue modules
Batra et al. (2016) [42]	Retinoblastoma *n* = 122 ^; sibling control group *n* = 50	* At study Mean (SD) 9.33 (0.3)	Surgery (*n* = 111) and/or XRT	27 (22)	XRT ^#^	Not reported	PedsQL Generic Core SR	Median (range) time from treatment: 4.5 (1–10.3) years	No difference in total scores between XRT and non-XRT group. XRT group total score is lower than control group
Netson et al. (2016) [27]	Various brain tumours (BT) *n* = 45; non-CNS cancer control (CC) *n* = 33; sibling control *n* = 36	Mean (SD) BT: 6.11 (3.45) CC: 3.36 (2.87)	BT: Surgery (STR 47%, NTR or GTR 53%) + XRT. Pre-XRT CT: 13% CC: does not receive CNS directed therapy	BT: 45 (100) CC: not reported	XRT Conformal or intensity-modulated	54 Gy (low grade glioma, craniopharyngioma, or ependymoma <18 months old at GTR); 59.4 Gy (ependymoma)	Kid KINDL-R: SR age 8-12; Kiddo KINDL-R: SR age 13-6; KINDL-R for Parents: PPR	Mean (SD) time from diagnosis: BT: 6.55 (2.52) years CC: 8.82 (3.66) years	BT cohort self-reported lower scores than the CC, but do not differ from sibling control. BT cohort is lower than sibling control on PPR, but did not differ from CC
An et al. (2011) [34] (2013) [35] *Direct overlap*	Various CNS tumours *n* = 31 [34] ^, *n* = 13 [35]; Age-matched control *n* = 125 [1]	Mean (SD) 8.67 (3.93) [34] 10.15 (2.58) [35]	Surgery + CT ± XRT ± peripheral blood SCT [34]. Protocol: Surgery, CT, XRT, CT, autologous stem cell rescue [35]	16 (51) [34] 13 (100) [35]	XRT ^#^ [34,35] CSI + boost to tumour bed [35]	23.4–30.6 Gy CSI and boost [35]	PedsQL Generic Core SR + PPR	Patients at different points during treatment regimen [34]. Cohort [2]: T1: During treatment; T2: mean (SD) of 12.69 (2.90) months post-T1 [35]	SR total score was lower than the control group [34]. No difference in total scores between T1 and T2 for SR and PPR [35]
Barrera et al. (2017) [36]	Various CNS tumours *n* = 91^	* At study. Mean (SD) 11.21 (2.76)	Surgery ± CT ± XRT	50 (55)	XRT ^#^ Whole brain *n* = 28; focal *n* = 22	Not reported	PedsQL Generic Core SR + PPR	T1: at study commencement (mean (SD) time since last treatment 4.06 (2.91) years) T2: 2 months post-T1 T3: 8 months post-T1	XRT had a negative impact on all SR scores. and physical, social, and school PPR scores. Total scores of all participants increased with time
Sato et al. (2014) [39]	Various CNS tumours *n* = 53	Mean (SD) 9.5 (4.1)	Surgery (85%), CT (75%), and/or XRT (79%)	42 (79)	XRT ^#^	Not reported	PedsQL Generic Core + subscales from brain tumour and cancer modules SR + PPR	Mean (SD) time from treatment: 4.6 (4.3) years	Moderate to high level agreement between SR + PPR
Penn et al. (2009) [44]	Various CNS tumours *n* = 35 ^	Median (range) 9.1 (1.5–16.4)	Surgery (details not provided), XRT (57%), and/or CT (31%)	20 (57)	XRT ^#^	Not reported	PedsQL Generic Core SR + PPR	T1: 1 month post-diagnosis T2: 6 months post-diagnosis T3: 12 months post-diagnosis	At T1, SR total score was lower for XRT than non-XRT group. No difference at any timepoint for PPR. SR + PPR scores increased from T1–T3
Dessens et al. (2016) [41]	Various CNS tumours *n* = 35	Median (range) 5.9 (0.1–13.8)	Surgery (87%), XRT (60%), and/or CT (54%)	21 (60)	XRT ^#^	Not reported	TACQOL SR + PPR	Median (range) time from diagnosis: 5.9 (1.8–11.0) years	Lower PPR scores than the normative data for motor and cognition scales, and lower SR scores for negative emotions scale
Musiol et al. (2019) [48]	Various CNS tumours *n* = 46 ^; age and sex matched control *n* = 104	Median (range) 6.5 (0.5–18.5)	Surgery (STR: *n* = 23, GTR *n* = 19), XRT (*n* = 32), and/or CT (*n* = 14)	32 (70)	XRT ^#^	Not reported	PedsQL Generic Core SR + PPR	Cross-sectional: median (range) time from treatment: 37 (3–123) months	SR + PPR scores were lower than the control group for all scales, excluding SR emotional functioning

Abbreviations: RT, radiation therapy; PROM, patient-reported outcome measure; SD, standard deviation; GTR, gross total resection; CT, chemotherapy; XRT, photon radiation therapy; CSI, craniospinal irradiation; PFB, posterior fossa boost; SR, self-report; PPR, parent–proxy report; HRQoL, health-related quality of life; PedsQL, Pediatric Quality of Life Inventory; EORTC, European Organisation for Research and Treatment of Cancer; T1–3, timepoint 1–3; SF-36, Short Form Health Survey; CNS, central nervous system tumour; IQR, inter-quartile range; STR, subtotal resection; NTR, near-total resection; KINDL-R, Kinder Lebensqualität fragebogen PROM; SCT, stem cell transplant; TACQOL, Netherlands Organisation for Applied Scientific Research Academic Medical Centre Children’s Quality of Life rating scale. ^#^ The radiation therapy modality is not reported. The authors determined that treatment was XRT from the availability of PRT in the study country and treating facility at date of treatment receipt (as identified on https://www.ptcog.ch/index.php/facilities-in-operation, last accessed 15 August 2022). ^ Please note, age and treatment details are only available for the larger sample (i.e., not just participants completing PROM or just participants with RT). * Age at diagnosis was not reported.

**Table 2:** Health-related quality of life in survivors of central nervous system tumours treated with proton radiation therapy.

Author	Diagnosis and Sample	Age at Diagnosis (Years)	Treatment Regimen	*n* (%) with RT	RT Modality and Technique	Prescribed Dose	PROM	Assessment Point(s)	Statistically Significant Outcomes
Badiyan et al. (2017) [30]	Low grade glioma *p* = 20 (18 treated at recurrence or progression) ^, *n* (completing PROM) = 16	Range 2.2–18.0	PRT ± surgery ± CT	16 (100)	PRT, PBS Focal 4# per week (2004–2007) 5# per week (2007–2014)	Mean (SD) dose delivered: 52.8 (7.1) Gy(RBE)	PEDQOL PPR	T1: baseline pre-PRT T2: 2 months post-PRT T3: 1 year post-PRT T4: 2 years post-PRT	No change in scores from any domain from T1–4
Weber et al. (2015) [32]	Non-metastatic atypical teratoid/rhabdoid tumour *p* = 15 ^, *n* (completing PROM) = 8	* Age at PRT median (range) 1.56 (0.38–2.28)	Surgery + PRT; pre or concurrent CT	15 (100)	PRT, spot scanning. Focal PRT. Sequential SFUD and intensity-modulated PRT delivery	54 Gy(RBE)	PedsQL Generic Core PPR	T1: pre-PRT T2: 2 months post-PRT	No change in PPR scores between T1 and T2
Kamran et al. (2018) [22]	Medulloblastoma *n* = 108 Primitive neuroectodermal tumour *n* = 8	Median (range) 7.6 (2.1–18.1)	Details of resection or CT not provided	116 (100)	PRT CSI + boost (tumour bed 74%, PFB 26%)	Not reported	PedsQL Generic Core SR + PPR	Baseline: average of 2–3 scores calculated during PRT. Ongoing annual assessment for median (range) of 5 (1–10.6) years	SR total score increased on average 1.8 points annually, PPR increased on average 2.0 points annually. At the last follow-up, SR physical score and all PPR subscales are worse than normative levels
Mouw et al. (2017) [23]	Retinoblastoma *p* = 12, *n* (completing PROM) = 9 ^	Median (range) 3 months (1–20 months)	Enucleation (*n* = 8), CT (*n* = 8), PRT, CT (*n* = 1)	9 (100)	PRT. Single lateral or posterior lateral field	Median dose: 44 Gy(RBE). Range: 40–48.6 Gy(RBE)	PedsQL Generic Core: PPR + SR for participants aged ≤ 17 18+ SR (*n* = 2): FACT-brain + FACT-fatigue	Median (range) length of follow up 12.9 years (5–22 years)	PedsQL scores were equal to the normative population
Kuhlthau et al. (2012) [24] + Yock et al. (2014) [25] *Direct overlap* *Potential overlap:* *Kamran n* = *50 [22]*	Various CNS tumours *p* = 142, *n* (completing PROM): ^ At T1 = 99 [24] At T5 = 57 [25]	Mean (range) 8.5 (2–18)	Surgery, CT, PRT (52.8%); surgery, PRT (31.0%); CT, PRT (9.2%); PRT only (7.0%)	142 (100)	PRT CSI + boost (43.0%); involved field PRT (57.0%)	95.8% received ≥ 45 Gy(RBE), 4.2% received <45 Gy(RBE)	PedsQL Generic Core, brain tumour and cancer modules SR + PPR	T1: During first week of PRT T2: During last 2 weeks of PRT T3: 1 year post-PRT T4: 2 year post-PRT T5: 3 year post-PRT	PPR total core scores increased from T1 to T5. All diagnoses, except for ‘other low-grade neoplasms’ saw increases in total core score from T1 to T5
Eaton et al. (2020) [26] *Overlap: Kuhlthau* *n* = *18 [24]*	Various CNS tumours *n* = 40; cross-sectional *n* = 22, longitudinal *n* = 18	* Age at PRT median (range) 2.5 (0.3–3.8)	CT (60%), high dose or intrathecal CT (25%)	40 (100)	PRT Supratentorial involved field (30%); infratentorial involved field (57.5%); CSI (12.5%)	Median (range) total dose: 54 Gy(RBE) (50.4–57.6 Gy(RBE)); CSI median dose: 23.4 Gy(RBE); CSI range: 18–36 Gy(RBE)	PedsQL Generic Core SR + PPR	Combined (*n* = 40) median (range) length of follow-up between treatment and last assessment: 6.7 (3–15.4) years. Longitudinal: T1: baseline pre-PRT T2: At final follow up median (range): 7.0 (3.1–11) years post-RT	All SR and PPR scores were lower than normative levels for all domains, excluding SR physical and school. PPR social functioning scores decreased from baseline to last follow up, with all other domains unchanged
Tran et al. (2020) [31] *Potential overlap: Badiyan n* = *16 [30] and Weber* *n* = *15 [32]*	Various CNS tumours *p* = 221 *n* (completing PROM) = 206 ^	Median (range) 3.1 (0.3-17.7)	Pre-PRT surgery (95%) ± CT (72.4%); concomitant CT: 17.2%	206 (100)	PRT, PBS focal PRT (200/221); CSI (21/221); partial XRT (7/221)	Median dose to PTV: 54 Gy(RBE); range 18–64.8 Gy(RBE)	PEDQOL SR + PPR if child > 5 years PedsQL Generic Core PPR if child < 5 years	T1: pre-PRT T2: 2 months post-PRT T3: 1 year post-PRT T4: 2 years post-PRT T5: 5 years post-PRT	PEDQOL PRR scores were below normative data, SR were above normative data. PedsQL PPR scores were below normative data

Abbreviations: RT, radiation therapy; PROM, patient-reported outcome measure; PRT, proton radiation therapy; CT, chemotherapy; PBS, pencil beam scanning; SD, standard deviation; PEDQOL, Quality of Life in Children and Adolescents with Cancer PROM; SR, self-report; PPR, parent–proxy report; T1–4, timepoint 1–4; SFUD, single-field uniform dose; PedsQL, Pediatric Quality of Life Inventory; CSI, craniospinal irradiation; PFB, posterior fossa boost; FACT, Functional Assessment of Cancer Therapy PROM; CNS, central nervous system tumour; XRT, photon radiation therapy. ^ Please note, age and treatment details are only available for the larger sample (i.e., not just participants completing PROM or just participants with RT). * Age at diagnosis was not reported.

#### 3.2.1. Influencing Factors

Three studies investigate the effect of socioeconomic status (SES) on the HRQoL of children treated for a CNS tumour with RT [22,26,43]. No effect is observed between SES and HRQoL, however variable methods of determining SES are used, and there is low representation of families within the low SES categories. Five studies find no statistically significant differences by sex in patients who undergo RT [22,24,26,43,48]. Three studies find no correlation between age and HRQoL [22,24,43], while Musiol et al. (2019) describe a weak negative correlation between age at diagnosis and self-reported emotional functioning [48]. Three studies analyse the impact of race, however there is limited representation of non-white participants that hinders producing a true analysis [22,24,26]. deMedeiros et al. (2020) find no difference in the overall HRQoL of survivors from Eastern and Western countries during a cross-sectional assessment, a mean of 6.58 years post-diagnosis [51].

One study compares the HRQoL of patients with craniopharyngioma following XRT or PRT [45]. At 1 and 3 years post-surgery, there is no statistically significant difference in HRQoL between the treatment modalities [45]. Due to the variability in patient demographics, assessment points and patient-reported outcome measures, a statistical comparison cannot be made between XRT and PRT outcomes for the cohort of CNS tumour studies.

Three studies found that the self-reported HRQoL is worse for patients with brain tumours requiring RT than patients not requiring RT [36,43,45]. Musiol et al. (2019) report that the overall self-reported HRQoL in survivors treated with both RT and chemotherapy is comparable with that of survivors treated only with chemotherapy [48].

The reporting of RT variables, such as prescribed dose and fractionation, treatment modality, and treatment field extent, is highly variable. Kamran et al. (2018) found that survivors who have a whole posterior fossa boost have greater improvements over time in parent–proxy reported HRQoL than survivors who have a tumour bed boost, but there is no differences between the self-reported HRQoL [22]. Kuhlthau et al. (2012) identify worse self-reported and parent–proxy reported HRQoL during the first week of PRT, for survivors who have craniospinal irradiation on one patient-reported outcome measure (PedsQL Generic Core), but no significant difference when using a different measure (PedsQL cancer module) [24]. Eaton et al. (2020) report no significant difference in parent–proxy reported HRQoL between patients who undergo craniospinal irradiation or involved field RT [26]. Kennedy et al. (2014) directly compare the HRQoL following standard and hyper-fractionated XRT, in matched cohorts, with no self-reported or parent–proxy reported differences at a median of 5.8 years after treatment [50]. In 11 of 30 studies, there are no details of prescribed dose and/or RT delivery technique, only the proportion of participants who received RT.

#### 3.2.2. Analysis Using PedsQL Generic Core Score

**Figure 2:**
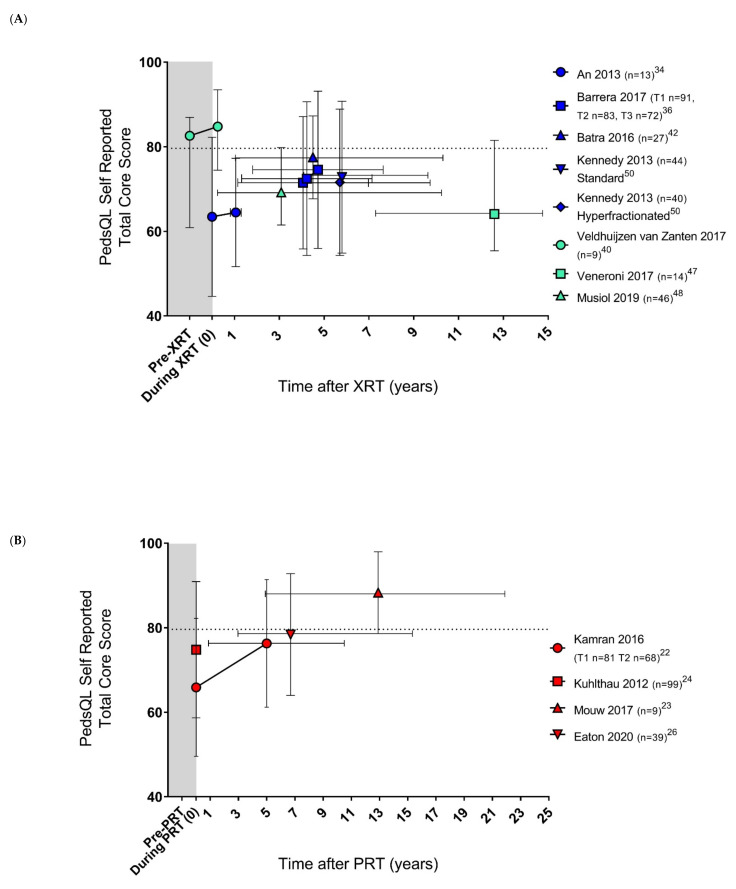

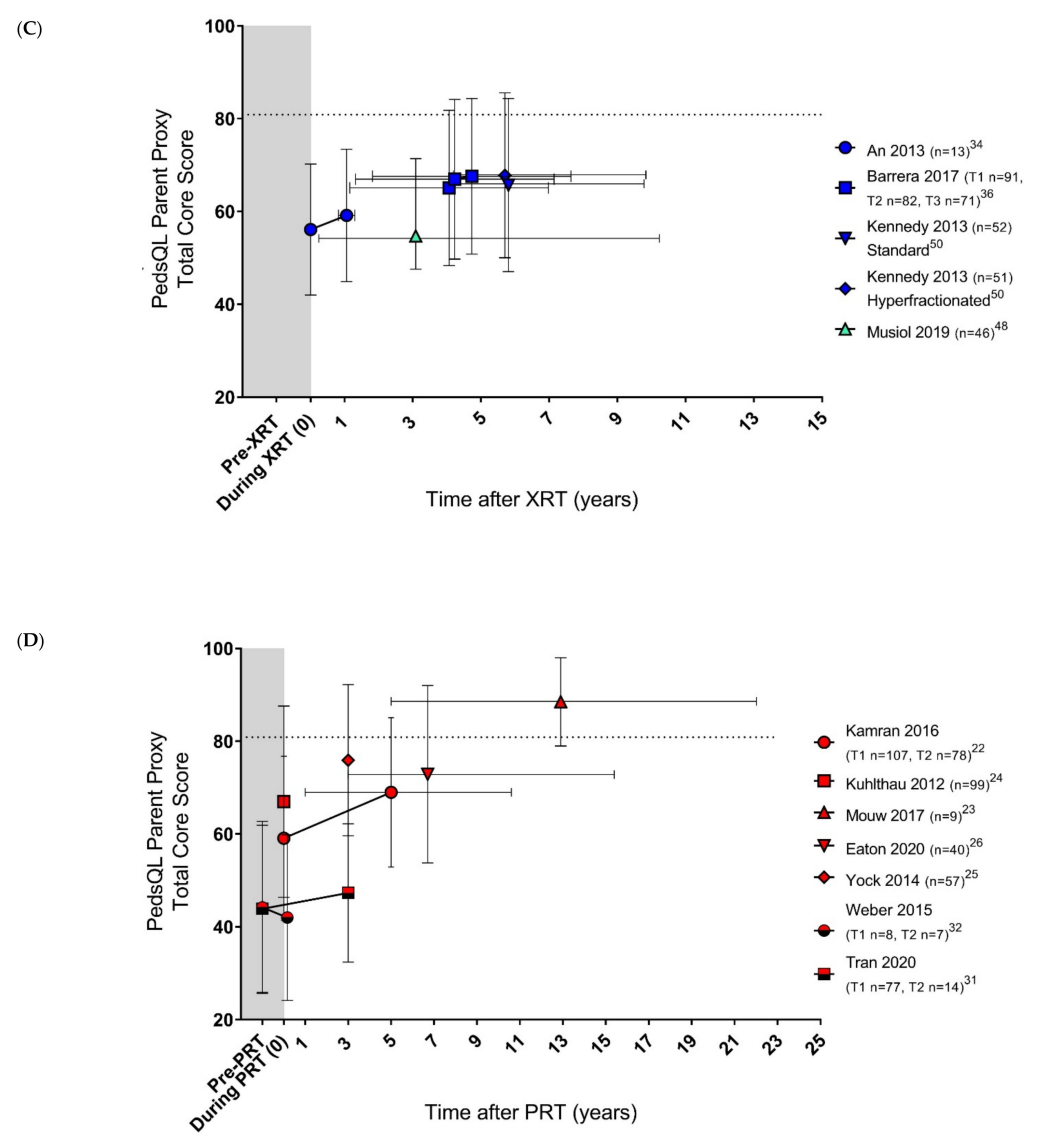
Health-related quality of life following photon (**A**,**C**) [34,36,40,42,47,48,50] and proton (**B**,**D**) [22,23,24,25,26,31,32] radiation therapy in paediatric central nervous system cancer, measured by PedsQL Generic Core self-report (**A**,**B**) and parent–proxy report (**C**,**D**). Legend. Blue: treated with photon radiation therapy. Mean and standard deviation (SD). Green: treated with photon radiation therapy. Median and inter-quartile ranges (IQR). Red: treated with proton radiation therapy. Mean and SD. The grey shading denotes assessment before or during treatment. Patients assessed during radiation therapy are placed at timepoint 0. Patients assessed prior to commencement of radiation therapy are placed at a combined timepoint before timepoint 0, due to each patient having a different duration from diagnosis to radiation therapy. Vertical lines represent SD or IQR. Horizontal lines denote duration of follow-up. The horizontal dashed line represents the mean healthy population PedsQL data from [52]. Higher PedsQL total core score indicates higher quality of life. Additional clarification on data was sought from [31]. Abbreviations: XRT, photon radiation therapy; PRT, proton radiation therapy; T, timepoint.

The PedsQL Generic Core score, version 4.0, was used in 22 of the total 30 studies. A total of 19 studies report the PedsQL total core score. Two studies are excluded for further analysis, as patients are described as ‘post diagnosis’, and could not definitively be placed on a timeline post-RT. Two studies include a directly overlapping cohort, so the study of highest quality assessment is included in our analysis. One study is excluded from analysis as it includes patients of both CNS and non-CNS tumour sites, and another is excluded as it is the only eligible PedsQL study describing a non-CNS tumour cohort. Figure 2 shows the self-reported and parent–proxy reported HRQoL following XRT and PRT CNS tumours based on the 14 eligible studies.

PedsQL HRQoL scores for survivors of a CNS childhood cancer appear to improve with increased time from treatment, however due to the inconsistency in measurement points and variable patient characteristics, statistical analysis and/or regression could not be performed to compare the outcomes of XRT and PRT. Of the studies that collected self-report and parent–proxy report outcomes for the same cohort, parent–proxy reports are lower than the child self-report, and further from the PedsQL normative population score.

### 3.3. Non-CNS Cancers

Three longitudinal and one cross-sectional study are identified with survivors treated for a non-CNS cancer (see Table 3) [33,37,46,49]. Harris et al. (2020) self-report the impact of multimodal treatment, including XRT, for chest wall sarcoma on the HRQoL of survivors a median of 5.5 years following diagnosis [49]. Leiser et al. (2016) parent–proxy report the HRQoL of patients with rhabdomyosarcoma of various locations, up to 2 years post-PRT [33]. Although differing patient-reported outcome measures are used, both sarcoma cohorts have comparable HRQoL to their respective normative reference populations. Two cohorts investigate HRQoL following XRT for Hodgkin disease [37,46]. One study shows self-reported increases in pain and xerostomia during treatment, but otherwise no significant changes in HRQoL during and acutely post-XRT [46]. Conversely, patients in the study by Klaassen et al. (2010) show an improvement in PedsQL Generic Core total score of HRQoL with time, during varied multimodal treatment [37].

Two studies combine several diagnoses (including CNS tumours, sarcomas, and blood cancers) into a single cohort for cross-sectional analyses (see Table 4) [29,38], however, clinical diversity of the cohorts, and differing lengths of follow-up, preclude further meaningful comparisons.

**Table 3:** Health-related quality of life in survivors of non-central nervous system tumours (photon and proton).

Author	Diagnosis and Sample	Age at Diagnosis (Years)	Treatment Regimen	*n* (%) with RT	RT Modality and Technique	Prescribed Dose	PROM	Assessment Point(s)	Statistically Significant Outcomes
Leiser et al. (2016) [33]	Rhabdomyosarcoma *p* = 83, *n* (completing PROM) = 34 ^	* Age at PRT range: 5–15.5	Surgery + PRT, pre or concurrent CT	34 (100)	PRT, PBS SFUD, intensity-modulated PRT, or both	Median dose: 54 Gy(RBE), range: 41.4–64.8 Gy(RBE)	PEDQOL PPR	T1: pre-PT T2: 2 months post-PRT T3: 1 year post-PRT T4: 2 years post-PRT	Scores for all domains (excluding cognition and social functioning with peers) increased from T1–T4. At T4, mean scores were comparable to the normative population
Harris et al. (2020) [49]	Chest wall sarcoma *p* = 175 ^, *n* (completing PROM) = 36	* Age at PROM completion median (IQR) 17.5 (14–22)	Surgery, CT, XRT: 42%; surgery, CT: 35%; CT, XRT: 13%	96 (55)	XRT Definitive XRT (*n* = 17). Timing: pre-operative (*n* = 9); post-operative (*n* = 66); pre + post-operative (*n* = 3)	Median (IQR) 50.4 Gy (41.3–56.0 Gy)	SR 8–18 years: PROMIS paediatric profile 37, v2. SR 18+ years: PROMIS 43, v2.1	Median (IQR) time from diagnosis: 5.5 (4.1–9) years	HRQoL was equivalent to the reference population in all domains, excluding anxiety, when all participants were combined
Marangoni-Lopes et al. (2016) [46]	Hodgkin disease *n* = 10 (location of involved area not reported) Matched control *n* = 10	Median (range) 13 (6–16)	CT + XRT	10 (100)	XRT	21.6 Gy	Portuguese version of Quality of Life—head and neck module (QLQ-H and N35) SR	T1: baseline T2: after 10.8 Gy T3: after 21.6 Gy (end of XRT) T4: 1 month post-XRT T5: 2 months post-XRT T6: 3 months post-XRT	Worsening in pain scores post-XRT from T1–T3. Worse xerostomia scores during RT than control group. No difference in other domains between participants and control group
Klaassen et al. (2010) [37]	Hodgkin disease *n* = 49 (location of involved area not reported)	Mean (range) 14.7 (8.9–18.0)	CT ± XRT	36 (73)	XRT ^#^	Not reported	SR, PPR and nurse proxy report: PedsQL Generic Core and cancer module, HUI 2 + 3, EuroQol EQ-5D	T1: 2 weeks after CT course 1 T2: 3rd day of CT course 2 T3: during the 3rd week of XRT T4: 1 year after diagnosis	90% of summary scores had at least moderate concordance with SR and PPR and/or nurse proxy report

Abbreviations: RT, radiation therapy; PROM, patient-reported outcome measure; PRT, proton radiation therapy; CT, chemotherapy; PBS, pencil beam scanning; SFUD, single-field uniform dose; PEDQOL, Quality of Life in Children and Adolescents with Cancer PROM; SR, self-report; PPR, parent–proxy report; T1–4, timepoint 1–4; IQR, inter-quartile range; XRT, photon radiation therapy; PROMIS, patient-reported outcome measurement information system PROM. ^#^ The radiation therapy modality is not reported. The authors determined that treatment was XRT from the availability of PRT in the study country and treating facility at date of treatment receipt (as identified on https://www.ptcog.ch/index.php/facilities-in-operation, last accessed 15 August 2022). ^ Please note, age and treatment details are only available for the larger sample (i.e., not just participants completing PROM or just participants with RT). * Age at diagnosis was not reported.

**Table 4:** Health-related quality of life in cohorts including multiple diagnoses or radiation therapy modalities.

Author	Diagnosis and Sample	Age at Diagnosis (Years)	Treatment Regimen	*n* (%) with RT	RT Modality and Technique	Prescribed Dose	PROM	Assessment Point(s)	Statistically Significant Outcomes
Eveslage et al. (2019) [45]	Craniopharyngioma *n* = 131 ^	Median (range) 9.7 (1.3–17.6)	Surgery ± RT 46 of 47 RT patients had an incomplete resection	47 (36)	XRT *n* = 22, PRT *n* = 22, other (seeds or stereotactic radiosurgery) *n* = 3	Not reported RT performed after progression *n* = 27	PEDQOL SR	T1: 1 year post-surgery T2: 3 years post-surgery	At T1 and T2, those who had undergone XRT had worse autonomy, body image and physical function than those who did not have any RT. No difference between PRT and those who did not have any RT. When directly compared, no difference between XRT and PRT scores
Ruccione et al. (2013) [29]	Total *n* = 94 ^ Leukaemia (*n* = 36), lymphoma (*n* = 23), CNS tumour (*n* = 9), soft tissue tumour (*n* = 19), bone tumour (*n* = 7)	* Age at XRT Mean (SD) 14.8 (2.74)	Surgery (*n* not reported) ± XRT (38%) ± CT (96%) ± SCT (5%)	36 (38)	XRT ^#^	Not reported	PedsQL Generic Core SR (only psychosocial summary score reported)	0–6 months post-XRT	Psychosocial summary score was lower for participants who had XRT than patients without XRT
Fukushima et al. (2017) [38]	Total *n* = 16 ^+^ Ependymoma (*n* = 3), Ewing sarcoma (*n* = 3), nasopharyngeal carcinoma (*n* = 3), germ cell tumour (*n* = 2), rhabdomyosarcoma (*n* = 2), parotid gland tumour (*n* = 1), neuroblastoma (*n* = 1), PNET (*n* = 1)	* Age at PRT median (range) 6.1 (2.4–13.7)	Variable surgery, CT, and/or SCT	16 (100)	PRT	Not reported separately from total eligible participants (*n* = 32). Median dose 54 Gy(RBE), range 19.8–78.4 Gy(RBE)	PedsQL Generic Core SR	Median (range) time from treatment: 5.2 (4.3–12.7) years	PedsQL scores were higher than Japanese population means for total core score and psychosocial summary score

Abbreviations: RT, radiation therapy; PROM, patient-reported outcome measure; XRT, photon radiation therapy; PRT, proton radiation therapy; PEDQOL, Quality of Life in Children and Adolescents with Cancer PROM; SR, self-report; T1–2, timepoint 1–2; SD, standard deviation; CT, chemotherapy; SCT, stem cell transplant; PedsQL, Pediatric Quality of Life Inventory. ^#^ The radiation therapy modality is not reported. The authors determined that treatment was XRT from the availability of PRT in the study country and treating facility at date of treatment receipt (as identified on https://www.ptcog.ch/index.php/facilities-in-operation, last accessed 15 August 2022). ^ Please note, age and treatment details are only available for the larger sample (i.e., not just participants completing PROM or just participants with RT). ^+^ Fukushima et al. 2017: 1 participant had PRT prior to 2000. Results were reported separately, so this participant was excluded from our analysis. * Age at diagnosis was not reported.

## 4. Discussion

To the best of our knowledge, this is the first systematic review identifying the patient-reported HRQoL following XRT and PRT for childhood cancer survivors treated after the year 2000. Based on the current evidence, a difference cannot be identified in HRQoL during or after RT, between XRT and PRT. This is a somewhat unexpected finding, and is likely due to the variability in patient characteristics, diagnoses, treatment regimens, and length of follow-up in the included studies of both modalities.

Five longitudinal studies report that HRQoL improves with time after treatment [22,24,33,37,44], whilst eight studies do not show a significant change [28,30,31,32,35,40,43,46]. Although several studies used PedsQL total core scores for survivors of a CNS tumour, a trend could not be identified, due to the diversity of tumour types, patient characteristics, applied therapies, and length of follow-up. There is minimal analysis of additional potential contributing factors such as age, sex, SES, and race in the studies included in this review. Younger age at radiation exposure has been associated with increased risk of toxicities [53], however, age does not appear to influence HRQoL outcomes for the participants within the included studies. Additionally, due to the lack of studies with baseline details, selection bias may have influenced the reported outcomes, as patients with poor prognosis, survival, and possibly worse quality of life, may have not been represented. However, poor prognosis may not always be an indicator of poor HRQoL, as shown by the two diffuse intrinsic pontine glioma cohorts reporting HRQoL appearing similar to, or higher than, normative PedsQL levels in the acute period post-XRT [28,40]. The outcomes of these cohorts may have been notably influenced by the small sample sizes (*n* = 25, *n* = 9) [28,40], and, therefore, a statistical comparison to the normative reference data with a large sample size is not reported.

The inconsistent reporting of RT details is a major limitation of the included studies. The severity of acute and late chronic effects of RT are partially related to the dose received by critical structures [54,55]. In particular, the dose received by brain substructures is correlated with critical neurocognitive outcomes [56], which may impact HRQoL. However, no studies meeting the inclusion criteria describe the association between irradiation of a specific organ or region and the associated HRQoL outcomes. Two studies examine the impact of prescribed dose on HRQoL, with no statistically significant outcomes [24,26]. Few studies consider the impact of an RT technique (e.g., craniospinal irradiation compared to focal treatment), or variable multimodal treatment regimens, however, baseline HRQoL data are not acquired. Without a baseline assessment, the difference in HRQoL could be due to confounding factors including pre-existing disease-specific causes, or additional intensive therapies. Therefore, it remains unknown what impact modern RT techniques may have on overall HRQoL outcomes. Armstrong et al. (2010) describe associations between region-specific doses to the brain and HRQoL in the Childhood Cancer Survivor Study cohort (1970–1986) [57]. Survivors who receive radiation exposure to temporal brain regions are at increased risk of memory and social functioning impairment [57]. With the now standard use of computed tomography-based dosimetry planning to accurately delineate brain substructures, reporting of dosimetric statistics and data describing outcomes produced by modulated RT techniques are required to expand on this knowledge. The authors recommend future HRQoL studies describing radiation oncology patients to follow Bentzen’s (1998) radiation oncology adaption of Consolidation of Standards for Reporting Trials (CONSORT) guidelines for reporting clinical outcome studies [58].

No studies report on the clinical utilisation or implementation of HRQoL assessments into routine practice. This is despite the growing body of evidence and calls for the integration of HRQoL assessment using patient-reported outcome measures into routine care to improve clinical outcomes and patient satisfaction [59,60,61,62]. Additionally, there are no details describing if patient-reported outcome measures were actioned by healthcare professionals, if they were deemed clinically significant. Notably, Bull et al. (2015) utilise the HRQoL outcomes presented in this review to identify a screening measure to detect cognitive deficit in children with cerebellar tumours, and conclude that PedsQL would be suitable for use in a clinical setting [43,63]. Therefore, routine clinical implementation of HRQoL assessment for paediatric patients would be an effective means to identify the impact of tumour and patient-specific factors prior to any therapeutic intervention, and to utilise HRQoL data to guide patient management, and acquire true baseline data to inform longitudinal studies. 

Another key solution to drive change will be an international effort to increase quality data collection following both XRT and PRT, and to promote data sharing to aid comparisons. This is underway and shown by the collaborative studies included in this review. Pediatric Proton Photon Consortium Registry and PanCareLIFE, are key examples of international consortia collecting longitudinal HRQoL outcomes for children with cancer, during and following XRT and PRT, to accompany clinical data [64,65]. Furthermore, the Children’s Oncology Group have recognised patient-reported HRQoL as an important metric by including PedsQL Generic Core assessment within their Standardised Neuropsychological and Behavioural Battery [66]. These efforts demonstrate the recognised importance of assessing patient-reported outcomes and perspectives to evaluate treatment options for children with cancer.

A potential limitation of this review is the inclusion of studies by the same institutions, with potentially overlapping patient cohorts. However, studies were not included in the analysis if they were estimated to have ≥50% of the same participants. This review does not include conference abstracts or grey literature, which may have described clinical action based upon HRQoL assessments, or provided further insight into potential routine assessment. Additionally, some studies may have been excluded during the screening processes by inadvertent human error or bias. Studies utilising ‘life functioning’ assessments were excluded because physical functioning may not be directly associated with the psychosocial domains of HRQoL, and do not consider the personal perspective of physical functioning on the individual’s HRQoL [67]. There is variability within the defined age ranges of ‘paediatric’ classification. Whilst some adolescents may be included in this review due to our upper age limit, we do not specifically synthesize the literature analysing outcomes for survivors of adolescent and young adult cancers. Due to the lack of robust evidence, this review is limited and could not analyse the difference between HRQoL during active treatment, acute follow-up, and long-term survivorship.

There is a large gap in the literature describing the HRQoL of non-CNS childhood cancer survivors following RT, and, hence, non-CNS diagnoses are underrepresented in this review. In countries where XRT is the standard of care, future research is needed to evaluate the HRQoL outcomes of XRT patients, particularly with intensity-modulated RT and volumetric-modulated arc therapy, to accompany the growing PRT evidence.

## 5. Conclusions

There is currently insufficient evidence to compare and determine if a difference exists between the HRQoL of children following XRT and PRT. There are limited rigorous HRQoL data following both XRT and PRT. This review highlights the importance of enhanced HRQoL collection, given the expanding global availability of PRT facilities, before concluding that PRT provides HRQoL improvements for this cohort. Standardised clinical implementation of HRQoL assessment using patient-reported outcomes may contribute to improvements in clinical care, and assist the rapid progression of knowledge comparing XRT and PRT. Improved reporting of prescribed dose and organ at risk dose constraints to accompany HRQoL assessments will assist in quantifying HRQoL outcomes for childhood cancer survivors following RT as treatment delivery techniques evolve.

## Supplementary Materials

The following supporting information can be downloaded at: https://www.mdpi.com/article/10.3390/cancers14163937/s1, Table S1: Search strategy for systematic review applied in Medline; Table S2: Quality assessment criteria table results.

## References

[1] Diller L., Chow E.J., Gurney J.G., Hudson M.M., Kadan-Lottick N.S., Kawashima T.I., Leisenring W.M., Meacham L.R., Mertens A.C., Mulrooney D.A. (2009). Chronic Disease in the Childhood Cancer Survivor Study Cohort: A Review of Published Findings. J. Clin. Oncol..

[2] Mahajan A., Stavinoha P.L., Rongthong W., Brodin N.P., McGovern S.L., El Naqa I., Palmer J.D., Vennarini S., Indelicato D.J., Aridgides P. (2021). Neurocognitive Effects and Necrosis in Childhood Cancer Survivors Treated with Radiation Therapy: A PENTEC Comprehensive Review. Int. J. Radiat. Oncol. Biol. Phys..

[3] Fakhry H., Goldenberg M., Sayer G., Aye S.S., Bagot K., Pi S., Ghazzaoui R., Vo N., Gowrinathan S., Bolton M. (2013). Health-Related Quality of Life in Childhood Cancer. J. Dev. Behav. Pediatr..

[4] Meadows A.T., Friedman D.L., Neglia J., Mertens A.C., Donaldson S.S., Stovall M., Hammond S., Yasui Y., Inskip P.D. (2009). Second Neoplasms in Survivors of Childhood Cancer: Findings from the Childhood Cancer Survivor Study Cohort. J. Clin. Oncol..

[5] Reimers T.S., Mortensen E.L., Nysom K., Schmiegelow K. (2009). Health-related quality of life in long-term survivors of childhood brain tumors. Pediatr. Blood Cancer.

[6] Krasin M.J., Constine L.S., Friedman D.L., Marks L.B. (2010). Radiation-Related Treatment Effects Across the Age Spectrum: Differences and Similarities or What the Old and Young Can Learn from Each Other. Semin. Radiat. Oncol..

[7] Anthony S.J., Selkirk E., Sung L., Klaassen R.J., Dix D., Scheinemann K., Klassen A. (2014). Considering quality of life for children with cancer: A systematic review of patient-reported outcome measures and the development of a conceptual model. Qual. Life Res..

[8] Leahy A.B., Steineck A. (2020). Patient-Reported Outcomes in Pediatric Oncology: The Patient Voice as a Gold Standard. JAMA Pediatr..

[9] Riedl D., Rothmund M., Darlington A.-S., Sodergren S., Crazzolara R., de Rojas T. (2021). Rare use of patient-reported outcomes in childhood cancer clinical trials—A systematic review of clinical trial registries. Eur. J. Cancer.

[10] Zientara N., Giles E., le Mbbs H., Short M. (2021). A scoping review of patient selection methods for proton therapy. J. Med. Radiat. Sci..

[11] Huynh M., Marcu L.G., Giles E., Short M., Matthews D., Bezak E. (2018). Current status of proton therapy outcome for paediatric cancers of the central nervous system—Analysis of the published literature. Cancer Treat. Rev..

[12] Eaton B.R., Macdonald S.M., Yock T.I., Tarbell N.J. (2015). Secondary Malignancy Risk Following Proton Radiation Therapy. Front. Oncol..

[13] Particle Therapy Co-Operative Group (2022). Particle Therapy Facilities in Clinical Operation. https://www.ptcog.ch/index.php/facilities-in-operation.

[14] Mizumoto M., Fuji H., Miyachi M., Soejima T., Yamamoto T., Aibe N., Demizu Y., Iwata H., Hashimoto T., Motegi A. (2021). Proton beam therapy for children and adolescents and young adults (AYAs): JASTRO and JSPHO Guidelines. Cancer Treat. Rev..

[15] Dell’Oro M., Short M., Wilson P., Hua C.-H., Gargone M., Merchant T., Bezak E. (2020). Influence of Target Location, Size, and Patient Age on Normal Tissue Sparing- Proton and Photon Therapy in Paediatric Brain Tumour Patient-Specific Approach. Cancers.

[16] Page M.J., McKenzie J.E., Bossuyt P.M., Boutron I., Hoffmann T.C., Mulrow C.D., Shamseer L., Tetzlaff J.M., Akl E.A., Brennan S.E. (2021). The PRISMA 2020 statement: An updated guideline for reporting systematic reviews. BMJ.

[17] Hardin A.P., Hackell J.M., Committee on Practice and Ambulatory Medicine (2017). Age Limit of Pediatrics. Pediatrics.

[18] Leduc A., Chaouni S., Pouzoulet F., de Marzi L., Megnin-Chanet F., Corre E., Stefan D., Habrand J.L., Sichel F., Laurent C. (2021). Differential normal skin transcriptomic response in total body irradiated mice exposed to scattered versus scanned proton beams. Sci. Rep..

[19] Kmet L.M., Lee R.C., Cook L.S. (2004). Standard Quality Assessment Criteria for Evaluating Primary Research Papers from a Variety of Fields.

[20] Coens C., Pe M., Dueck A.C., Sloan J., Basch E., Calvert M., Campbell A., Cleeland C., Cocks K., Collette L. (2020). International standards for the analysis of quality-of-life and patient-reported outcome endpoints in cancer randomised controlled trials: Recommendations of the SISAQOL Consortium. Lancet Oncol..

[21] Varni J.W. (2022). The PedsQL Measurement Model for the Pediatric Quality of Life Inventory—About the Model. http://www.pedsql.org/about_pedsql.html.

[22] Kamran S.C., Goldberg S.I., Kuhlthau K.A., Lawell M.P., Weyman E.A., Gallotto S.L., Hess C.B., Huang M.S., Friedmann A.M., Abrams A.N. (2018). Quality of life in patients with proton-treated pediatric medulloblastoma: Results of a prospective assessment with 5-year follow-up. Cancer.

[23] Mouw K.W., Yeap B.Y., Caruso P., Fay A., Misra M., Sethi R.V., MacDonald S.M., Chen Y.-L., Tarbell N.J., Yock T.I. (2017). Analysis of patient outcomes following proton radiation therapy for retinoblastoma. Adv. Radiat. Oncol..

[24] Kuhlthau K.A., Pulsifer M.B., Yeap B.Y., Morales D.R., Delahaye J., Hill K.S., Ebb D., Abrams A.N., MacDonald S.M., Tarbell N.J. (2012). Prospective study of health-related quality of life for children with brain tumors treated with proton radiotherapy. J. Clin. Oncol..

[25] Yock T.I., Bhat S., Szymonifka J., Yeap B.Y., Delahaye J., Donaldson S.S., MacDonald S.M., Pulsifer M.B., Hill K.S., DeLaney T.F. (2014). Quality of life outcomes in proton and photon treated pediatric brain tumor survivors. Radiother. Oncol..

[26] Eaton B.R., Goldberg S., Tarbell N.J., Lawell M.P., Gallotto S.L., Weyman E.A., Kuhlthau K.A., Ebb D.H., MacDonald S.M., Yock T.I. (2020). Long-term health-related quality of life in pediatric brain tumor survivors receiving proton radiotherapy at <4 years of age. Neuro-Oncology.

[27] Netson K.L., Ashford J.M., Skinner T., Carty L., Wu S., Merchant T.E., Conklin H.M. (2016). Executive dysfunction is associated with poorer health-related quality of life in pediatric brain tumor survivors. J. Neurooncol..

[28] Mandrell B.N., Baker J., Levine D., Gattuso J., West N., Sykes A., Gajjar A., Broniscer A. (2016). Children with minimal chance for cure: Parent proxy of the child’s health-related quality of life and the effect on parental physical and mental health during treatment. J. Neurooncol..

[29] Ruccione K., Lu Y., Meeske K. (2013). Adolescents’ psychosocial health-related quality of life within 6 months after cancer treatment completion. Cancer Nurs..

[30] Badiyan S.N., Ulmer S., Ahlhelm F.J., Fredh A.S.M., Kliebsch U., Calaminus G., Bolsi A., Albertini F., Leiser D., Timmermann B. (2017). Clinical and Radiologic Outcomes in Adults and Children Treated with Pencil-Beam Scanning Proton Therapy for Low-Grade Glioma. Int. J. Part. Ther..

[31] Tran S., Lim P.S., Bojaxhiu B., Teske C., Baust K., Zepter S., Kliebsch U., Timmermann B., Calaminus G., Weber D.C. (2020). Clinical outcomes and quality of life in children and adolescents with primary brain tumors treated with pencil beam scanning proton therapy. Pediatr. Blood Cancer.

[32] Weber D.C., Ares C., Malyapa R., Albertini F., Calaminus G., Kliebsch U., Mikroutsikos L., Morach P., Bolsi A., Lomax T. (2015). Tumor control and QoL outcomes of very young children with atypical teratoid/rhabdoid tumor treated with focal only chemo-radiation therapy using pencil beam scanning proton therapy. J. Neurooncol..

[33] Leiser D., Calaminus G., Malyapa R., Bojaxhiu B., Albertini F., Kliebsch U., Mikroutsikos L., Morach P., Bolsi A., Walser M. (2016). Tumour control and Quality of Life in children with rhabdomyosarcoma treated with pencil beam scanning proton therapy. Radiother. Oncol..

[34] An K.J., Song M.S., Sung K.W., Joung Y.S. (2011). Health-related quality of life, activities of daily living and parenting stress in children with brain tumors. Psychiatry Investig..

[35] An K.J., Joung Y.S., Sung K.W., Kim J.H. (2013). Health-related quality of life and cognitive functioning at on- and off-treatment periods in children aged between 6-13 years old with brain tumors: A prospective longitudinal study. Yonsei Med. J..

[36] Barrera M., Atenafu E.G., Schulte F., Bartels U., Sung L., Janzen L., Chung J., Cataudella D., Hancock K., Saleh A. (2017). Determinants of quality of life outcomes for survivors of pediatric brain tumors. Pediatr. Blood Cancer.

[37] Klaassen R.J., Barr R.D., Hughes J., Rogers P., Anderson R., Grundy P., Ali S.K., Yanofsky R., Abla O., Silva M. (2010). Nurses provide valuable proxy assessment of the health-related quality of life of children with Hodgkin disease. Cancer.

[38] Fukushima H., Fukushima T., Suzuki R., Iwabuchi A., Hidaka K., Shinkai T., Masumoto K., Muroi A., Yamamoto T., Nakao T. (2017). Comorbidity and quality of life in childhood cancer survivors treated with proton beam therapy. Pediatr. Int..

[39] Sato I., Higuchi A., Yanagisawa T., Murayama S., Kumabe T., Sugiyama K., Mukasa A., Saito N., Sawamura Y., Terasaki M. (2014). Impact of late effects on health-related quality of life in survivors of pediatric brain tumors. Cancer Nurs..

[40] Veldhuijzen van Zanten S.E.M., El-Khouly F.E., Jansen M.H.A., Bakker D.P., Sanchez Aliaga E., Haasbeek C.J.A., Wolf N.I., Zwaan C.M., Vandertop W.P., van Vuurden D.G. (2017). A phase I/II study of gemcitabine during radiotherapy in children with newly diagnosed diffuse intrinsic pontine glioma. J. Neurooncol..

[41] Dessens A.B., van Herwerden M.C., Aarsen F.K., Birnie E., Catsman-Berrevoets C.E. (2016). Health-related quality of life and emotional problems in children surviving brain tumor treatment: A descriptive study of 2 cohorts. Pediatr. Hematol. Oncol..

[42] Batra A., Ma D.D., Paul R., Patekar M., Dhawan D., Bakhshi S. (2016). Quality of Life Assessment in Retinoblastoma: A Cross-Sectional Study of 122 Survivors from India. Pediatr. Blood Cancer.

[43] Bull K.S., Liossi C., Culliford D., Peacock J.L., Kennedy C.R. (2014). Child-related characteristics predicting subsequent health-related quality of life in 8-to 14-year-old children with and without cerebellar tumors: A prospective longitudinal study. Neuro-Oncol. Pract..

[44] Penn A., Lowis S.P., Stevens M.C., Hunt L.P., Shortman R.I., McCarter R.J., Pauldhas D., Curran A.L., Sharples P.M. (2009). Family, demographic and illness-related determinants of HRQL in children with brain tumours in the first year after diagnosis. Pediatr. Blood Cancer.

[45] Eveslage M., Calaminus G., Warmuth-Metz M., Kortmann R.-D., Pohl F., Timmermann B., Schuhmann M.U., Flitsch J., Faldum A., Müller H.L. (2019). The Postoperative Quality of Life in Children and Adolescents with Craniopharyngioma. Dtsch. Arztebl. Int..

[46] Marangoni-Lopes L., Rodrigues L., Mendonça R., Nobre-Dos Santos M. (2016). Radiotherapy changes salivary properties and impacts quality of life of children with Hodgkin disease. Arch. Oral Biol..

[47] Veneroni L., Boschetti L., Barretta F., Clerici C.A., Simonetti F., Schiavello E., Biassoni V., Spreafico F., Gandola L., Pecori E. (2017). Quality of life in long-term survivors treated for metastatic medulloblastoma with a hyperfractionated accelerated radiotherapy (HART) strategy. Childs Nerv. Syst..

[48] Musiol K., Bulska W., Brozek P., Oslizlo B., Ryzak S., Dubiel J., Sobol-Milejska G. (2019). Quality of life in survivors of childhood brain tumour and the association of children’s diseases on quality of their parents life. Psychooncology.

[49] Harris C.J., Helenowski I., Murphy A.J., Mansfield S.A., LaQuaglia M.P., Heaton T.E., Cavalli M., Murphy J.T., Newman E., Overmen R.E. (2020). Implications of Tumor Characteristics and Treatment Modality on Local Recurrence and Functional Outcomes in Children with Chest Wall Sarcoma: A Pediatric Surgical Oncology Research Collaborative Study. Ann. Surg..

[50] Kennedy C., Bull K., Chevignard M., Culliford D., Dörr H.G., Doz F., Kortmann R.-D., Lannering B., Massimino M., Gutiérrez A.N. (2014). Quality of survival and growth in children and young adults in the PNET4 European controlled trial of hyperfractionated versus conventional radiation therapy for standard-risk medulloblastoma. Int. J. Radiat. Oncol. Biol. Phys..

[51] De Medeiros C.B., Moxon-Emre I., Scantlebury N., Malkin D., Ramaswamy V., Decker A., Law N., Kumabe T., Leonard J., Rubin J. (2020). Medulloblastoma has a global impact on health related quality of life: Findings from an international cohort. Cancer Med..

[52] Varni J.W., Seid M., Kurtin P.S. (2001). PedsQL™ 4.0: Reliability and Validity of the Pediatric Quality of Life Inventory™ Version 4.0 Generic Core Scales in Healthy and Patient Populations. Med. Care.

[53] Dell’Oro M., Short M., Wilson P., Bezak E. (2021). Normal tissue tolerance amongst paediatric brain tumour patients-current evidence in proton radiotherapy. Crit. Rev. Oncol. Hematol..

[54] Wang K., Tepper J.E. (2021). Radiation therapy-associated toxicity: Etiology, management, and prevention. CA Cancer J. Clin..

[55] Palmer J.D., Tsang D.S., Tinkle C.L., Olch A.J., Kremer L.C., Ronckers C.M., Gibbs I.C., Constine L.S. (2021). Late effects of radiation therapy in pediatric patients and survivorship. Pediatr. Blood Cancer.

[56] Acharya S., Guo Y., Patni T., Li Y., Wang C., Gargone M., Ashford J.M., Wilson L., Faught A., Reddick W.E. (2022). Association Between Brain Substructure Dose and Cognitive Outcomes in Children with Medulloblastoma Treated on SJMB03: A Step Toward Substructure-Informed Planning. J. Clin. Oncol..

[57] Armstrong G.T., Jain N., Liu W., Merchant T.E., Stovall M., Srivastava D.K., Gurney J.G., Packer R.J., Robison L.L., Krull K.R. (2010). Region-specific radiotherapy and neuropsychological outcomes in adult survivors of childhood CNS malignancies. Neuro-Oncology.

[58] Bentzen S.M. (1998). Towards evidence based radiation oncology: Improving the design, analysis, and reporting of clinical outcome studies in radiotherapy. Radiother. Oncol..

[59] Bele S., Chugh A., Mohamed B., Teela L., Haverman L., Santana M.J. (2020). Patient-Reported Outcome Measures in Routine Pediatric Clinical Care: A Systematic Review. Front. Pediatr..

[60] Engelen V., Detmar S., Koopman H., Maurice-Stam H., Caron H., Hoogerbrugge P., Egeler R.M., Kaspers G., Grootenhuis M. (2012). Reporting health-related quality of life scores to physicians during routine follow-up visits of pediatric oncology patients: Is it effective?. Pediatr. Blood Cancer.

[61] Schepers S., Engelen V., Haverman L., Caron H., Hoogerbrugge P., Kaspers G., Egeler R., Grootenhuis M. (2014). Patient reported outcomes in pediatric oncology practice: Suggestions for future usage by parents and pediatric oncologists. Pediatr. Blood Cancer.

[62] Meryk A., Kropshofer G., Hetzer B., Riedl D., Lehmann J., Rumpold G., Haid A., Holzner B., Crazzolara R. (2021). Implementation of daily patient-reported outcome measurements to support children with cancer. Pediatr. Blood Cancer.

[63] Bull K.S., Liossi C., Peacock J.L., Yuen H.M., Kennedy C.R., Children’s Cancer and Leukaemia Group (2015). Screening for cognitive deficits in 8 to 14-year old children with cerebellar tumors using self-report measures of executive and behavioral functioning and health-related quality of life. Neuro-Oncology.

[64] Lawell M.P., Indelicato D.J., Paulino A.C., Hartsell W., Laack N.N., Ermoian R.P., Perentesis J.P., Vatner R., Perkins S., Mangona V.S. (2020). An open invitation to join the Pediatric Proton/Photon Consortium Registry to standardize data collection in pediatric radiation oncology. Br. J. Radiol..

[65] Calaminus G., Baust K., Berger C., Byrne J., Binder H., Casagranda L., Grabow D., Grootenhuis M., Kaatsch P., Kaiser M. (2021). Health-Related Quality of Life in European Childhood Cancer Survivors: Protocol for a Study Within PanCareLIFE. JMIR Res. Protoc..

[66] Embry L., Annett R.D., Kunin-Batson A., Patel S.K., Sands S., Reaman G., Noll R.B. (2012). Implementation of multi-site neurocognitive assessments within a pediatric cooperative group: Can it be done?. Pediatr. Blood Cancer.

[67] Shelly A., Davis E., Waters E., MacKinnon A., Reddihough D., Boyd R., Reid S., Graham H.K. (2008). The relationship between quality of life and functioning for children with cerebral palsy. Dev. Med. Child Neurol..

