# Supplementary material for: Can We Compare the Health-Related Quality of Life of Childhood Cancer Survivors Following Photon and Proton Radiation Therapy? A Systematic Review
Source: Cancers (Basel). 2022 Aug 15;14(16):3937. doi: 10.3390/cancers14163937 (PMC9405962; doi:10.3390/cancers14163937)
Supplement: Supplementary file 1 [file cancers-14-03937-s001.zip › cancers-1870055-supplementary.pdf]

Supplementary Table S1. Search strategy for systematic review applied in Medline.

1.	exp Child/
2.	Pediatrics/
3.	(child* or pediatric* or paediatric*).mp.
4.	1 or 2 or 3
5.	exp Neoplasms/
6.	(cancer* or tumor* or tumour* or oncolog* or neoplas* or carcinoma* or malignan*).mp.
7.	5 or 6
8.	(quality of life or QoL or survivorship or wellness).mp.
9.	"Quality of Life"/
10.	8 or 9
11.	patient reported outcome measures/
12.	(patient reported outcome* or patient reported outcome measure* or self report* or self-report* or self-rate or self rate* or proxy-rate* or proxy or child report* or child-report* or parent-proxy or questionnaire* or survey* or validated instrument*).mp.
13.	11 or 12
14.	exp Radiotherapy/
15.	(proton* or proton therapy or proton beam therapy or radiation or radiation therapy or radiotherapy or X-ray therapy or xray therapy or particle therapy or chemoradiotherapy or external beam).mp.
16.	14 or 15
17.	4 and 7 and 10 and 13 and 16
18.	limit 17 to (english language and yr="2000 -Current")

Supplementary Table S2. Quality assessment criteria table results

Criteria	Yes (%)	Partial (%)	No (%)
1. Question/objective sufficiently described?	**100.0**	**0.0**	**0.0**
2. Study design evident and appropriate?	**90.0**	**10.0**	**0.0**
3. Method of subject/comparison group selection or source of information/input variables described and appropriate?	**93.3**	**6.7**	**0.0**
4. Subject (and comparison group, if applicable) characteristics sufficiently described?	**73.4**	**23.3**	**3.3**
5. If interventional and random allocation was possible, was it described?	n/a	n/a	n/a
6. If interventional and blinding of investigators was possible, was it reported?	n/a	n/a	n/a
7. If interventional and blinding of subjects was possible, was it reported?	n/a	n/a	n/a
8. Outcome and (if applicable) exposure measure(s) well defined and robust to measurement/misclassification bias? Means of assessment reported?	**55.0**	**41.7**	**3.3**
9. Sample size appropriate?	**21.7**	**43.3**	**35.0**
10. Analytic methods described/justified and appropriate?	**68.3**	**15.0**	**16.7**
11. Some estimate of variance is reported for the main results?	**80.0**	**16.7**	**3.3**
12. Controlled for confounding?	**28.3**	**41.7**	**30.0**
13. Results reported in sufficient detail?	**50.0**	**35.0**	**15.0**
14. Conclusions supported by the results?	**96.7**	**3.3**	**0.0**

Assessment of criterion were informed by agreement with SISAQOL recommendations. Sufficient sample size was defined as 50 or more participants. Partial sufficiency of sample size was 30-50 participants. Insufficient sample size was defined as below 30 participants.
